# New Appliances and Things Medical

**Published:** 1907-08-31

**Authors:** 


### NEW APPLIANCES AND THINGS MEDICAL.

[We shall be glad to receive at our Office, 28 & 29, Southampton Street, Strand, London, W.C., from the manufacturers, specimens of all new preparations and appliances which may be brought out from time to time.]

### ALLENBURYS' MILK FOOD COCOA.

(Allen and Hanburys, Limited, 37 Lombard Street, London, E.C.)

The sample forwarded is a preparation of pure cocoa extract, deprived of a greater part of fatty matter, and "Allenburys' Peptonised Milk Food." Made according to the directions enclosed with the sample—that is to say, simply like any ordinary cocoa, with boiling water—it forms a highly nutritious beverage, which in point of flavour compares favourably with the best cocoas on the market. The absence of superfluous fats, altogether apart from the fact that the addition of peptonised milk renders it especially digestible for the convalescent, and the retention of the pure flavour of unsophisticated cocoa, should win it the approval of those who desire an easily made and withal an agreeable and digestible beverage. In using this milk cocoa the addition of milk is, of course, entirely unnecessary, but by slightly increasing the quantity of cocoa and adding a spoonful of cream an excellent chocolate may be prepared with it, equal in most respects to chocolate made in the approved fashion from many other cocoa preparations.

### A HYGIENIC COIN PAD.

Mr. H. Thornton, of 31 Warwick Road, Margate, has forwarded for our inspection a model of his patent coin pad and hygienic finger damper, as designed for confectioners' use. The practice of damping the finger with the saliva is unfortunately too commonly observed, and anything that can easily obviate this method of securing a grip with the finger pad is worthy of approval. Mr. Thornton's model consists of a porcelain pad into which is fitted a piece of corrugated cork and an absorbent pad—the damper. Underneath the whole is a string box. The model is exceedingly strongly made, and forms an attractive counter ornament, and it may be utilised in many ways. As an advertising medium it has obvious advantages, and as it is simple, strong, and hygienic it is an arrangement which we should like to see in common use in all shops. It is an invention which is worth wider publicity than it appears so far to have received.

### HORSFORD'S ACID PHOSPHATE.

(Rumford Chemical Works, Providence, U.S.A.)

We have received a sample of Horsford's Acid Phosphate, which consists of a solution of the phosphates of lime, magnesia, potash, and iron with phosphoric acid. These drugs have long had a reputation as valuable tonics. In the East a dilute solution of phosphoric acid has been used for many centuries as an aphrodisiac, and is used to this day. The tonic action of the phosphates is especially directed to the nervous system, of which they and the so-called organic phosphates form an integral part. There are also many conditions of malnutrition in which the phosphates undoubtedly exert a beneficial action, because they not only provide in themselves nutritive elements, but also enable the body to make better use of the other nutritive elements contained in food. There has of recent years been very considerable scientific controversy with regard to the relative merits of the inorganic phosphates to which the preparation before us belongs and the organic phosphates, such, for instance, as lecithin and its allies. Recent research has certainly not shaken the inorganic phosphates which have the advantage of being much less expensive than their more complicated allies. Horsford's Acid Phosphate is an exceedingly pleasant and suitable form of inorganic phosphate, and we think the reputation it has earned is entirely justified.

### LOCAL ANÆSTHETICS.

(Bengué and Co. London Office, 91 Great Titchfield Street, London, W.)

This firm has forwarded three varieties of their ethyl chloride preparations. Of these "Narcotile" is a very pure chloride of ethyl, prepared by a special process. The absence of impurities and of any strong, unpleasant odour is a valuable point in its favour, and makes it one of the best preparations of this valuable anæsthetic on the market. "Ænestile" is a preparation of pure ethyl chloride, mixed with a small amount of methyl choride, which probably enhances its action as a local anæsthetic. The firm also supplies a pure preparation of ethyl chloride, which we have found to act excellently both for local and general anæsthetic purposes. All these preparations are put up in specially patented tubes, fitted with a valve stopper of simple and convenient construction. With a little practice it is easy for the operator to work the valve with his forefinger and to regulate to a nicety the amount of anæsthetic administered, according to the graduated scale on each tube.

### ABSORBENT COTTON FIBRE.

(T. Syjer and Co., 45 Wilson Street, Finsbury Square, London, E.C.)

This is natural cotton fibre, undyed, and, so far as we have been able to find, without any impurity, if we except a small amount of dust from which no specimen can long be kept free at this season of the year in a London office. The fibres are long and free from knots. Such a material is easily sterilisable by dry heat, and, owing to its softness, it may be used instead of gauze in dressings. The specimen submitted was remarkably absorbent, both of watery and of heavier fluids such as blood serum. The cotton fibre is supplied in 1 cwt. bags and in smaller 7 lb. parcels, at a comparatively cheap price, considering its purity.

### HALL'S WASHABLE DISTEMPER.

(Messrs. Sisson Brothers and Co., Ltd., Hull.)

The class of water paints known as "distempers" is one of which many varieties are on the market, but experience of the often inartistic effects some of these paints produce on the walls and brickwork coated with them convinces us that the majority do not come up to the standard of perfection claimed by their makers. There are a few exceptions, and the most striking of these is undoubtedly the washable distemper manufactured by Messrs. Sisson Brothers. It is an extremely quick-drying paint, and portions of unglazed porcelain painted with it showed no alteration in any way after having been vigorously scrubbed with a weak lysol solution. That is a test which the majority of so-called washable paints will not survive. Applied according to the directions—that is, simply mixed with water—the paint gives a smooth, lustrous surface, which does not readily peel off or crack when exposed to the sun. The hygienic advantages of such a medium are undoubtedly great, and for tropical climates such a paint should prove a veritable boon and a blessing. We are less willing, however, to agree that the small amount of cresylic acid which the specimen submitted contained can have any decided mucor-destroying power. The antiseptic value of the distemper is small, but walls coated with it can be rendered more or less aseptic, and for hospitals and sick rooms that is an advantage which it is not easy to over-estimate. The paint is supplied in a variety of shades, and the makers issue a neat little pamphlet, "Modern Development in House Decoration," which shows the manifold ways in which it may be applied to advantage in beautifying the house.

